# The Validity of Predictive Equations to Estimate 24-Hour Sodium Excretion: The MESA and CARDIA Urinary Sodium Study

**DOI:** 10.1093/aje/kwx056

**Published:** 2017-06-09

**Authors:** Norrina B. Allen, Lihui Zhao, Catherine M. Loria, Linda Van Horn, Chia-Yih Wang, Christine M. Pfeiffer, Mary E. Cogswell, Jacqueline Wright, Kiang Liu

**Keywords:** blood pressure, epidemiologic methods, hypertension, sodium, validation

## Abstract

We examined the population distribution of urinary sodium concentrations and the validity of existing equations predicting 24-hour sodium excretion from a single spot urine sample among older adults with and without hypertension. In 2013, 24-hour urine collections were obtained from 554 participants in the Multi-Ethnic Study of Atherosclerosis and the Coronary Artery Risk Development in Young Adults study, who were aged 45–79 years and of whom 56% were female, 58% were African American, and 54% had hypertension, in Chicago, Illinois. One-third provided a second 24-hour collection. Four timed (overnight, morning, afternoon, and evening) spot urine specimens and the 24-hour collection were analyzed for sodium and creatinine concentrations. Mean 24-hour sodium excretion was 3,926 (standard deviation (SD), 1,623) mg for white men, 2,480 (SD, 1,079) mg for white women, 3,454 (SD, 1,651) mg for African-American men, and 3,397 (SD, 1,641) mg for African-American women, and did not differ significantly by hypertensive status. Mean bias (difference) in predicting 24-hour sodium excretion from the timed spot urine specimens ranged from −182 (95% confidence interval: −285, −79) to 1,090 (95% confidence interval: 966, 1,213) mg/day overall. Although the Tanaka equation using the evening specimen produced the least bias overall, no single equation worked well across subgroups of sex and race/ethnicity. A single spot urine sample is not a valid indicator of individual sodium intake. New equations are needed to accurately estimate 24-hour sodium excretion for older adults.

Higher levels of urinary sodium excretion, and potentially low levels as well, have been associated with an increased risk of hypertension, cardiovascular events, and mortality (1–6). It has been estimated that worldwide up to 1.65 million deaths from cardiovascular disease per year can be attributed to excess sodium consumption (7). However, many of these studies have relied upon single spot urine samples to represent an individual's sodium intake despite little validation of these equations and recent work questioning their validity across populations. Accurate assessment of sodium intake is essential for evaluating the association between individual sodium intake and cardiovascular disease outcomes and for monitoring changes in population sodium intake over time. The gold-standard method of assessment of sodium intake among individuals is derived from measures of 24-hour urinary sodium excretion with multiple samples needed to account for within-individual day-to-day variability (8); however, the collection process is burdensome for study participants, resulting in decreased participation and incomplete collection, and it can be expensive for large, population-based studies.

Recently researchers have used the sodium excreted in a single spot urine sample to estimate both individual and population sodium intake (9, 10), despite questions regarding the validity of this method due to diurnal and circadian variations in sodium excretion, particularly among diverse racial groups (11–13). Although some studies suggest a single spot urine specimen may be adequate for evaluating mean sodium excretion among some groups (14, 15), differential bias exists in individual estimates across the distribution of sodium excretion. Using a single spot urine specimen, actual sodium excretion is overestimated at lower sodium levels and underestimated at higher sodium levels even among young adults without hypertension (14, 16, 17). Little is known about how the potential bias at the individual and group level might vary among older individuals, those with hypertension, and African Americans, in whom differences in the pattern of diurnal urine sodium excretion may exist (11).

The goal of the current study was to examine the validity of 4 published predictive equations to determine how well single, timed spot urine specimens can estimate an individual's 24-hour urinary sodium excretion among African-American and white men and women of older age groups (45–79 years) and those with hypertension. Thus, we first determined the population mean level of 24-hour sodium excretion as measured among participants of the Multi-Ethnic Study of Atherosclerosis (MESA) and Coronary Artery Risk Development in Young Adults (CARDIA) Urinary Sodium Study, and we then determined how well the timed spot urine samples estimated the 24-hour urine sodium excretion among older African-American men and women. Last, we examined the relative validity of existing predictive equations using spot urine sodium concentrations for predicting 24-hour urine sodium excretion in older adults and those with hypertension, and whether the relative validity varied by the timing of the timed spot urine samples.

## METHODS

### Participants

We recruited participants involved in the MESA, MESA Family, and CARDIA studies who resided in the Chicago area to participate in this study. Details of the MESA (18) and CARDIA (19–21) studies have been published previously. We included men and women between of ages 45–79 years, who were white or African American and had no evidence of severe kidney disease (estimated glomerular filtration rate (eGFR) was ≥30). Of the 1,130 eligible participants, 574 (51%) consented to participate in the study and attended the exam. Participants had similar demographic characteristics compared with the eligible nonparticipants (Web Table 1, available at https://academic.oup.com/aje). Sixteen participants had incomplete 24-hour urine collections, and 4 were deemed ineligible. A total of 554 participants completed the exam (96%) and at least one 24-hour urine collection. Recruitment occurred from March 15, 2013, through August 15, 2013. The study was approved by the site's institutional review board, and all participants provided informed consent.

### Study design

Data for this study included sociodemographic characteristics, medication use (participants were asked to bring with them all medications they were currently taking), height and weight, blood pressure (defined as the average of the second and third blood pressure readings), dietary assessment (using the Automated Self-Administered 24-hour Recall (ASA24)) (22), blood draw to determine eGFR using serum creatinine, and at least 1 fractional 24-hour urine collection, begun in the clinic. Each participant completed at least one 24-hour urine collection and one-third were randomly sampled to complete a second 24-hour urine collection 4–14 days later. Similar to the Centers for Disease Control and Prevention (CDC) study (23), collections were considered completed if the total urine volume was greater than or equal to 500 mL, women did not report menstruation at any time during collection, the length of collection was greater than 20 hours, and there were no reports of spilling urine or missing a void more than once during the collection. From among the voids during each 24-hour collection, 4 timed-spot urine samples were selected, representing the overnight (first void after the longest period of sleep and between 04:00 am and 12:00 noon), morning (first void between 08:30 am and 12:30 pm), afternoon (first void between 12:31 pm and 5:30 pm), and evening (first void between 5:31 pm and 11:59 pm) times that corresponded to the CDC study among younger adults. A composite 24-hour sample was derived by taking a proportional aliquot from each void. A 1-mL aliquot was taken from this composite and from each of the 4 timed-spot urine specimens. All vials were shipped frozen on dry ice to the CDC's National Center for Environmental Health for analysis. The concentrations of sodium and creatinine were measured using ion-selective electrodes and the Cobas ISE/Na^+^ assay on the Hitachi Modular P clinical analyzer (Roche Diagnostics Corp., Indianapolis, Indiana). Two different levels of quality control samples were analyzed together with the study samples at the beginning and end of each analytic run. As in the previous CDC study, the between-run measurement imprecision was ≤3% (23).

Hypertension was defined according to clinical guidelines as blood pressure (BP) greater than 140/90 mm Hg and/or on BP–lowering medications (24). Individuals were considered to have controlled BP if they reported taking an antihypertensive medication and their BPs were less than 140/90 mm Hg. If BP was greater than 140/90 mm Hg, this was considered uncontrolled. Antihypertensive medications were grouped by class such that diuretics could be examined separately.

### Statistical analysis

We calculated the amount of each analyte (sodium (Na) and creatinine) by multiplying the analyte concentration by the corresponding volume of the specimen. The volume for the 24-hour urine collection was adjusted for the self-reported collection time (that is, as (total volume/total collection time in hours) × 24). We examined the mean and standard deviation of each analyte overall and within subgroups according sex, race/ethnicity, and hypertension status. Statistical tests of differences between subgroups were done using *t* tests. We used 2 methods to examine the day-to-day variability of the analytes. Within-person coefficient of variation was calculated among the subset of participants with two 24-hour urine collections as the square root of the within-person variance divided by the mean of each analyte. We also examined the day-to-day variability by the ratio of within-person to between-persons variances.

We evaluated the ability of the 4 predictive equations (International Cooperative Study on Salt and Blood Pressure (INTERSALT), Tanaka, Kawasaki, and Mage) (Web Table 2) to estimate population mean levels of 24-hour sodium excretion from single spot urine specimens (25–29). These equations include spot sodium concentration, sex, creatinine, potassium, height, weight, and age. We first examined the means and standard deviations for the measured and the predicted 24-hour sodium excretion using each of the 4 equations and each of the 4 timed spot urine specimens, except for the Kawasaki equation, which was developed specifically to be used with a morning sample. Means were examined overall and by sex, race/ethnicity, and hypertension status. We calculated bias as the difference between the predicted and measured 24-hour sodium excretion. The statistical significance of the difference was assessed by the paired *t* test. The group mean bias was examined by equation and timing of spot sample (overnight, morning, afternoon, evening) overall and by sex, race/ethnicity, and hypertension status subgroup. We plotted the group mean bias along with the 95% confidence intervals (30, 31). The 95% confidence intervals were calculated as within 1.96 × standard error of the mean differences.

Individual biases were skewed, and so Bland-Altman relative bias plots were used to describe the agreement between the estimated 24-hour sodium excretions from each of the 4 timed spot specimens and the measured 24-hour sodium excretion (30, 31). The Bland-Altman relative bias is the individual difference between the predicted and the measured sodium excretion divided by the mean of the predicted and measured sodium. To assess the difference in individual bias across the range of 24-hour sodium excretion, we plotted the relative bias for each individual against the mean of the individual's predicted and measured sodium and determined the 95% limits of agreement (relative bias within 1.96 × standard deviation). We also calculated Spearman correlation coefficients and examined whether individual bias differed across the range of 24-hour sodium excretion. Analyses were conducted overall and according to sex and race/ethnicity.

Several sensitivity analyses were conducted to determine whether the completeness of the samples affected our findings. First, we repeated the analyses including only individuals who reported not missing any voids and not spilling or missing any urine at any void (*n* = 442). Second, to address the potential bias from incomplete samples, we restricted our analysis to individuals who had a measured-to-expected 24-hour urine creatinine ratio of ≥0.6 based on equations for expected (predicted) creatinine using age, weight, height, and race/ethnicity (*n* = 541) (28). In addition, we stratified our analyses by hypertension status, use of diuretics, and eGFR (≤60 and >60). *P* values < 0.05 were considered statistically significant. All analyses were conducted using SAS, version 9.4 (SAS Institute, Inc., Cary, North Carolina), and R (R Foundation for Statistical Computing, Vienna, Austria).

## RESULTS

Among the 554 participants, the average age was 60 years, 56% were female, and 58% were African American (Table 1). Just over half of all participants had hypertension, and the average body mass index was 30 (calculated as weight (kg)/height (m)^2^). On the day prior to urine collection, participants consumed an average of 2,088 kcal and 3,470 mg of sodium. On average each participant collected 9 voids (range, 3–23; Web Table 3). The mean total urine volume was 1,838 mL, and only 2% of participants reported missing a void. A second 24-hour urine collection was completed by 186 (33.6%) participants. Participants who completed a second 24-hour urine collection had demographic characteristics and risk factors similar to all participants.

**Table 1.** Characteristics of Study Participants, Chicago, Illinois, 2013

Characteristic	Total			Participants Who Completed 2 Days of Collection			Participants Who Completed Only 1 Day of Collection		
	No. of Participants	%	Mean (SD)	No. of Participants	%	Mean (SD)	No. of Participants	%	Mean (SD)
Sample size^a^	554			186			368		
Female	310	56.0		103	55.4		207	56.25	
Age, years			60.3 (9.1)			60.1 (9.2)			60.4 (9.1)
African-American	319	57.6		111	59.7		208	56.5	
Hypertension	283	51.0		93	50.0		190	51.6	
Diuretic users	88	15.9		35	18.8		53	14.4	
BMI^b^			30.0 (6.5)			30.6 (6.6)			29.8 (6.4)
Day 1 dietary intake									
Energy, kcal/day			2,088.5 (908.9)			2,092.7 (835.8)			2,086.4 (945.0)
Sodium, g/day			3,470.2 (1,906.7)			3,452.9 (1,677.3)			3,479.0 (2,015.4)

Abbreviations: BMI, body mass index; SD, standard deviation.

^a^ Missing 2 participants for BMI: total, *n* = 552; among those who completed 2 days of collection, *n* = 184. Missing 2 participants for dietary intake: total, *n* = 552; among those who completed 2 days of collection, *n* = 186.

^b^ BMI was calculated as weight (kg)/height (m)^2^.

### Urine analyte excretions

The total urine volume was greatest for white men, followed by white women (Web Table 4). White men excreted over 3,900 milligrams of sodium within the 24-hour collection, higher than all other race/ethnicity and sex groups. However, the amount of creatinine excreted was similar for African-American and white men and was higher than for women regardless of race (Web Table 5). Sodium and creatinine concentrations appeared to be highest in the evening spot specimens.

### Within-person variance between collections on day 1 and day 2

One-third of this sample (*n* = 186) completed a second 24-hour urine collection. Among these individuals the day-to-day variation in 24-hour sodium ranged from 31% to 70% and in creatinine ranged from 24% to 51% (Web Table 6). Variation was larger for African Americans than for whites for all analytes. Variances were larger for the timed spot specimens, although there were no consistent patterns across analyte or race/ethnicity.

The ratio of within-person to between-persons variance for 24-hour sodium was 1.5 for African Americans and 1.1 for whites. The ratio for within-person to between-persons variance was larger for the timed spot specimens and generally larger among African Americans.

### Measured versus predicted 24-hour sodium excretions

The measured and predicted sodium excretion varied by timing, sex, and race/ethnicity (Table 2). Overall, the absolute mean bias was smallest for the Tanaka equation with the evening specimen (61 mg/day, 95% confidence interval (CI): −43, 165) and greatest for the Kawasaki equation using the morning specimen (1,089 mg/day, 95% CI: 966, 1,213) (Web Figure 1).

**Table 2.** Mean Values and Standard Deviations for 24-Hour Sodium Excretion by Population Subgroup and the Timing^a^ of Spot Urine Collection Among Adults Aged 45–79 Years, Measured and Predicted According to Different Prediction Equations, Chicago, Illinois, 2013

Subgroup and Timing	No. of Participants	Measured 24-Hour Sodium Excretion, mg/day	Equation and Predicted 24-Hour Sodium Excretion, mg/day			
			INTERSALT	Tanaka	Kawasaki	Mage
All						
Overnight	540	3,273 (1,536)	3,182 (922)	3,468 (840)		3,352 (2,553)
Morning	529	3,292 (1,546)	3,188 (966)	3,614 (971)	4,381 (1,481)	3,801 (2,942)
Afternoon	520	3,302 (1,538)	3,166 (972)	3,540 (924)		3,579 (2,723)
Evening	537	3,291 (1,539)	3,108 (986)	3,352 (871)		3,118 (2,273)
African-American men						
Overnight	131	3,407 (1,609)	3,811 (838)	3,392 (864)		3,639 (2,532)
Morning	124	3,419 (1,628)	3,885 (840)	3,547 (856)	4,559 (1,389)	4,031 (2,774)
Afternoon	119	3,492 (1,576)	3,887 (775)	3,558 (744)		3,944 (2,277)
Evening	128	3,415 (1,621)	3,714 (946)	3,281 (856)		3,375 (2,370)
African-American women						
Overnight	179	3,326 (1,473)	2,854 (674)	3,546 (909)		3,870 (3,051)
Morning	180	3,348 (1,478)	2,889 (749)	3,701 (1,045)	4,276 (1,503)	4,387 (3,534)
Afternoon	170	3,369 (1,495)	2,861 (745)	3,660 (1,073)		4,274 (3,482)
Evening	181	3,344 (1,478)	2,811 (750)	3,395 (908)		3,458 (2,464)
White men						
Overnight	108	3,933 (1,617)	3,873 (634)	3,569 (742)		3,153 (1,771)
Morning	105	3,963 (1,635)	3,827 (643)	3,760 (904)	4,863 (1,470)	3,708 (2,354)
Afternoon	108	3,927 (1,624)	3,867 (597)	3,676 (763)		3,467 (2,012)
Evening	109	3,933 (1,617)	3,806 (666)	3,534 (764)		3,144 (1,857)
White women						
Overnight	122	2,474 (1,082)	2,390 (611)	3,346 (774)		2,461 (2,077)
Morning	120	2,490 (1,087)	2,354 (707)	3,426 (1,000)	3,937 (1,414)	2,760 (2,264)
Afternoon	123	2,475 (1,087)	2,268 (686)	3,235 (928)		2,359 (1,967)
Evening	119	2,494 (1,082)	2,279 (714)	3,196 (895)		2,305 (2,021)

Abbreviation: INTERSALT, International Cooperative Study on Salt and Blood Pressure.

^a^ The timing of urine specimens was as follows: overnight (first void after the longest period of sleep and between 04:00 am and 12:00 noon), morning (first void between 08:30 am and 12:30 pm), afternoon (first void between 12:31 pm and 5:30 pm), and evening (first void between 5:31 pm and 11:59 pm).

**Figure 1.**
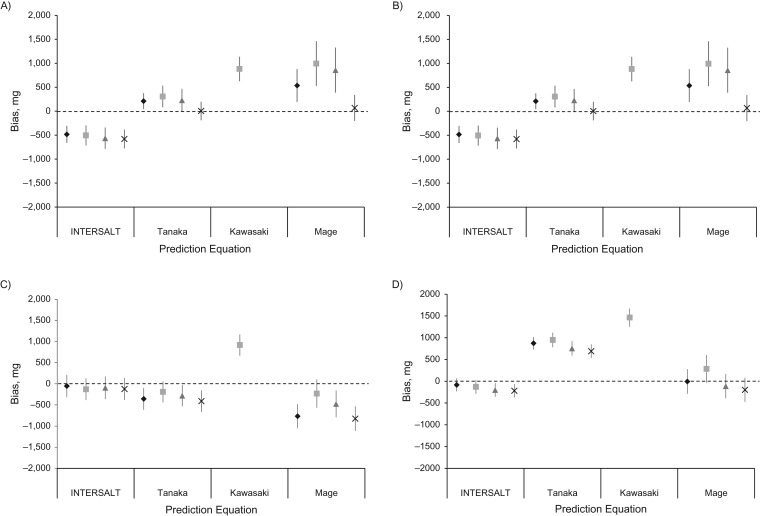
Mean bias in predicted minus measured 24-hour urinary sodium excretion based on the same day according to prediction equation and according to timing of the spot urine collection among adults aged 45–79 years, Chicago, Illinois, 2013. Results are shown for the following race/ethnicity and sex subgroups: African-American men (*n* = 134) (A); African-American women (*n* = 185) (B); white men (*n* = 110) (C); and white women (*n* = 125) (D). The mean bias in predicted 24-hour sodium excretion by using each equation is shown for a single urine specimen collected overnight (♦), in the morning (■), in the afternoon (▲), and in the evening (×). Note that the Kawasaki equation was developed specifically to be used with a morning sample. INTERSALT, International Cooperative Study on Salt and Blood Pressure. Bars, 95% confidence intervals.

Across all race/ethnicity and sex subgroups, except African-American women, the Kawasaki equation using the morning specimen consistently had the highest amount of mean bias, overestimating sodium excretion by 877–1,089 mg/day (Figure 1). In contrast, within race/ethnicity and sex subgroups, the equation with the least amount of bias varied. For African-American men, the Tanaka equation using the overnight specimen provided the least amount of absolute bias, and, using the Tanaka equation, the biases for all of the timed spot specimens were nonsignificant. For African-American women, the Tanaka equation using the evening specimen produced the least bias (51 mg/day, 95% CI: −118, 220) and yet was not significant. For white men, the Tanaka equation significantly underestimated sodium excretion. Instead, the INTERSALT equation produced the least amount of bias, especially when using the overnight (−59 mg/day, 95% CI: −320, 202) and afternoon (−60 mg/day, 95% CI: −326, 206) specimens. For white women, the bias was not significant for the INTERSALT equation using the overnight and morning specimens; however, the Mage equation using the overnight specimen produced the least bias for white women (−12 mg/day, 95% CI: −295, 270). For white women, the Mage equation produced no statistically significant bias using any timed spot specimen, although it underestimated sodium excretion among white men and overestimated it among African-American men and women.

In a sensitivity analysis, when the least biased sex- and race/ethnicity-specific equation was used, the amount of bias in the overall population was reduced, and lowest when using the overnight sample (Web Table 7).

### Relative individual differences in predicted and measured 24-urinary sodium excretion

**Figure 2.**
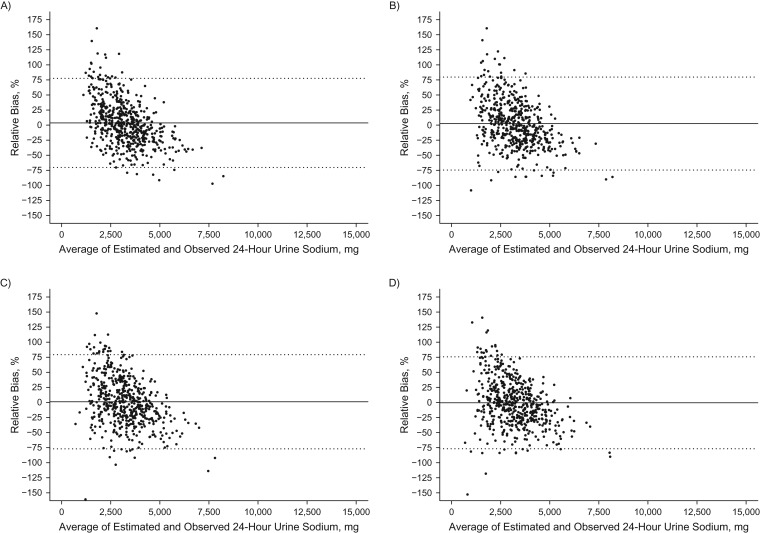
Bland-Altman plots of the relative bias (difference in agreement) between measured 24-hour urinary sodium excretion on one day and predicted 24-hour urinary sodium excretion on the same day based on the International Cooperative Study on Salt and Blood Pressure (INTERSALT) equation (25) and spot urinary sodium among adults aged 45–79 years, Chicago, Illinois, 2013. Samples were collected overnight (*n* = 553) (A), in the morning (*n* = 540) (B), in the afternoon (*n* = 528) (C), and in the evening (*n* = 545) (D). The relative bias for each individual is the predicted minus the measured 24-hour sodium excretion divided by the mean of the predicted and measured 24-hour urinary sodium multiplied by 100, and it is plotted against the mean of the predicted and measured 24-hour sodium excretion. The solid black line represents the mean relative difference (bias). Dashed lines, 95% limits of agreement of the mean relative difference within 1.96 × standard deviation.

**Figure 3.**
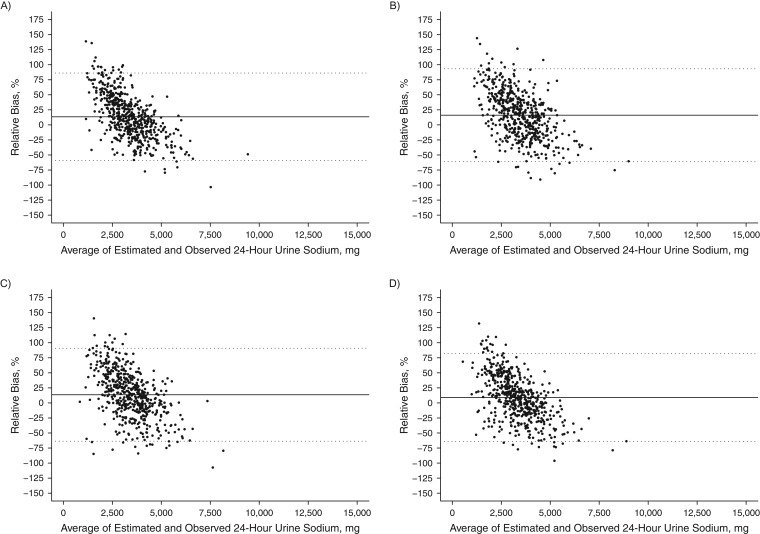
Bland-Altman plots of the relative bias (difference in agreement) between measured 24-hour urinary sodium excretion on one day and predicted 24-hour sodium excretion on the same day based on the Tanaka equation (26) and spot urinary sodium among adults aged 45–79 years, Chicago, Illinois, 2013. Samples were collected overnight (*n* = 553) (A), in the morning (*n* = 540) (B), in the afternoon (*n* = 528) (C), and in the evening (*n* = 545) (D). The relative bias for each individual is the predicted minus the measured 24-hour sodium excretion divided by the mean of the predicted and measured 24-hour urinary sodium multiplied by 100, and it is plotted against the mean of the predicted and measured 24-hour sodium excretion. The solid black line represents the mean relative difference (bias). Dashed lines, 95% limits of agreement of the mean relative difference within 1.96 × standard deviation.

**Figure 4.**
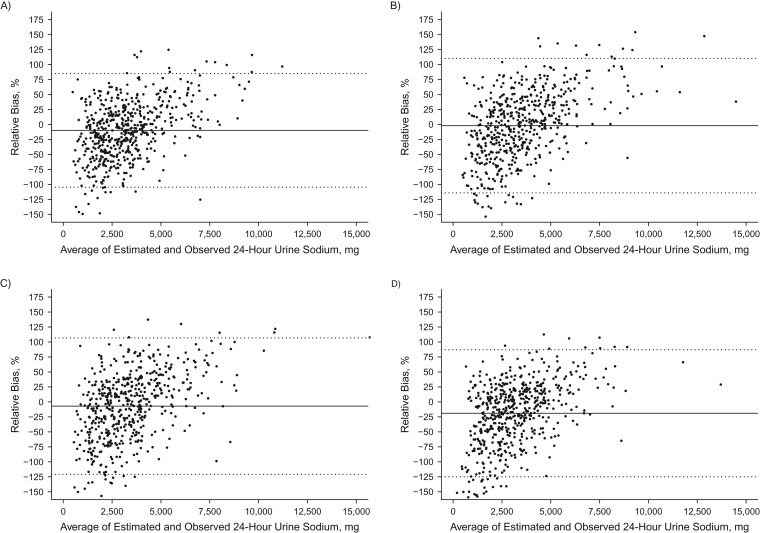
Bland-Altman plots of the relative bias (difference in agreement) between measured 24-hour sodium excretion on one day and predicted 24-hour sodium excretion on the same day based on the Mage equation (28) and spot urinary sodium among adults aged 45–79 years, Chicago, Illinois, 2013. Samples were collected overnight (*n* = 553) (A), in the morning (*n* = 540) (B), in the afternoon (*n* = 528) (C), and in the evening (*n* = 545) (D). The relative bias for each individual is the predicted minus the measured 24-hour sodium excretion divided by the mean of the predicted and measured 24-hour urinary sodium multiplied by 100, and it is plotted against the mean of the predicted and measured 24-hour sodium excretion. The solid black line represents the mean relative difference (bias). Dashed lines, 95% limits of agreement of the mean relative difference within 1.96 × standard deviation.

**Figure 5.**
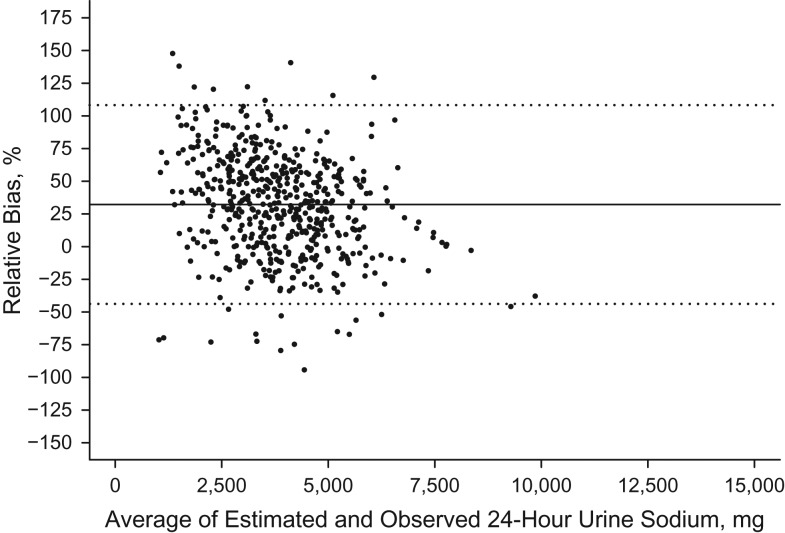
Bland-Altman plot of the relative bias (difference in agreement) between measured 24-hour sodium excretion on one day and predicted 24-hour sodium excretion on the same day based on the Kawasaki equation (27) and spot urinary sodium among adults aged 45–79 years, Chicago, Illinois, 2013. The sample was the morning specimen (*n* = 540). The relative bias for each individual is the predicted minus the measured 24-hour sodium excretion divided by the mean of the predicted and measured 24-hour urinary sodium multiplied by 100, and it is plotted against the mean of the predicted and measured 24-hour sodium excretion. The solid black line represents the mean relative difference (bias). Dashed lines, 95% limits of agreement of the mean relative difference within 1.96 × standard deviation.

Among individuals, bias between the predicted and measured 24-hour urinary sodium excretion was large (95% limits of agreement across equations, −124.9% to +110.2%) and varied by the level of sodium excretion. For both the INTERSALT and Tanaka equations, lower sodium levels were positively biased while higher sodium levels were negatively biased (Figures 2 and 3). The opposite pattern was seen with the Mage equation (Figure 4). The Kawasaki equation produced the highest amount of average relative bias (32.2%, 95% CI: 32.1, 32.4), although the individual bias appeared to be less skewed across the distribution of 24-hour sodium excretion (Figure 5).

### Individual correlations with measured 24-hour sodium excretion

The correlations of individual predicted 24-hour sodium excretion with measured 24-sodium excretion were moderate to high (Table 3). For all participants, the Mage equation was the most highly correlated with measured 24-hour sodium excretion and ranged from 0.57 to 0.68. Correlation coefficients varied across race/ethnicity and sex subgroups and timed spot specimens. Predicted 24-hour urinary sodium excretion was most highly correlated with the overnight specimens using each of the 4 equations. In general, for the overnight and evening specimens, the Mage equation showed the highest level of agreement, as shown by intraclass correlation coefficients in Web Table 8.

**Table 3.** Individual Correlation Coefficients (*r*)^a^ With Measured 24-Hour Sodium Excretion, by Population Subgroup and Timing^b^ of Spot Urine Specimen Collections Among Adults Aged 45–79 Years, Chicago, Illinois, 2013

Subgroup and Timing	No. of Participants	Measured Sodium Excreted in Timed Spot Specimen	Equation and Predicted 24-Hour Sodium Excretion			
			INTERSALT	Tanaka	Kawasaki	Mage
All						
Overnight	540	0.60	0.62	0.62		0.68
Morning	529	0.51	0.57	0.53	0.55	0.59
Afternoon	520	0.50	0.55	0.51		0.57
Evening	537	0.59	0.62	0.61		0.66
African-American men						
Overnight	131	0.63	0.67	0.64		0.67
Morning	124	0.46	0.54	0.54	0.55	0.55
Afternoon	119	0.42	0.47	0.42		0.44
Evening	128	0.60	0.64	0.62		0.64
African-American women						
Overnight	179	0.53	0.58	0.61		0.63
Morning	180	0.44	0.47	0.41	0.40	0.46
Afternoon	170	0.38	0.49	0.42		0.49
Evening	181	0.53	0.57	0.56		0.60
White men						
Overnight	108	0.62	0.56	0.57		0.67
Morning	105	0.67	0.67	0.63	0.65	0.70
Afternoon	108	0.62	0.55	0.59		0.65
Evening	109	0.51	0.55	0.58		0.64
White women						
Overnight	122	0.55	0.58	0.63		0.69
Morning	120	0.41	0.59	0.59	0.58	0.64
Afternoon	123	0.45	0.54	0.55		0.60
Evening	119	0.43	0.55	0.57		0.61

Abbreviation: INTERSALT, International Cooperative Study on Salt and Blood Pressure.

^a^ All values are Spearman correlation coefficients between measured 24-hour sodium excretion and all sodium in a timed spot urine specimen or predicted 24-hour sodium excretion based on sodium concentration from a spot urine collection used with 1 of the 4 estimation equations.

^b^ The timing of urine specimens was as follows: overnight (first void after the longest period of sleep and between 04:00 am and 12:00 noon), morning (first void between 08:30 am and 12:30 pm), afternoon (first void between 12:31 pm and 5:30 pm), and evening (first void between 5:31 pm and 11:59 pm). All correlation coefficients were statistically significant at *P* < 0.05.

### Sensitivity analyses

When analyses were restricted to the 98% of participants who reported that they had not missed any voids, findings were the same. Additionally, there were no meaningful differences when we excluded those with a measured-to-expected 24-hour urine creatinine ratio of <0.6. In stratified analyses, we obtained similar findings regardless of eGFR or whether the participant had been diagnosed and/or treated for hypertension, regardless of blood pressure control (Web Figures 2–6). About 16% (*n* = 88) of participants were taking diuretics at the time of urine collection. When these individuals were excluded, mean 24-hour sodium excretions were 3,934 for white men, 3,325 for African-American men, 2,526 for white women, and 3,355 mg for African-American women, and the accuracy of spot specimens was relatively unchanged.

## DISCUSSION

In this study of older individuals, many with hypertension, we found that using any of the 4 previously published equations to estimate 24-hour urinary sodium from a single timed spot urine specimen resulted in significant over- or underestimation of 24-hour sodium excretion at the group level. No single published equation produced unbiased sodium estimates for all 4 sex-race/ethnicity subgroups, and in fact the group-level bias produced by each equation varied according to specimen timing, sex, and race/ethnicity but not hypertension status, eGFR, or use of diuretics. Although the absolute levels of bias were relatively small, the variation in magnitude and direction of the bias with race/ethnicity and sex are very important when comparing differences in sodium excretion among these subgroups.

In our study, the Tanaka equation using the evening sample produced the least bias in the overall sample but overestimated the sodium excretion of white women and underestimated it in white men. In race/ethnicity- and sex-specific results, the Tanaka equation appeared to produce the least bias when using either the afternoon or evening specimens among African Americans, whereas among whites the INTERSALT equation using the overnight or morning specimens produced no significant bias. In contrast, there was no difference in race/ethnicity- and sex-specific analyses in the CDC study, which found that the INTERSALT equation provided the least-biased estimation for all younger individuals (17). For white women, the Mage equation using the overnight sample produced the least absolute bias. The reasons for these differences remain unclear and may be results of racial/ethnic and sex differences in eating patterns and/or salt retention. Further, as with young healthy adults, individual-level bias in estimated 24-hour sodium excretion, as shown in the Bland-Altman plots, varied greatly and appeared to change from low to high sodium levels despite moderate correlations with 24-hour sodium excretion.

Our findings are in contrast to the recent publication by Mente et al. (15), in which the Kawasaki equation was found to produce the least group-level bias. Differences in study findings may be due to the timing of the urine specimen used in the Kawasaki equation and differences in the ranges of 24-hour sodium excretion. The spot urine specimens used by Mente et al. were collected as the “first urine pass the following morning” after 24-hour collection, which is closer to the “overnight” specimen collection in the present study. Mente et al. tested all 3 formulae, including the Kawasaki equation, with this specimen. In the present study, the Kawasaki equation was applied only to the morning specimens, because that is considered to be most comparable to the original Kawasaki specimen, which was described as “Urine collected at approximately 8:00 am, before breakfast but after the urine collection at the rising time the next morning, was also used as a spot urine specimen, which is tentatively called a second morning voiding urine specimen (SMU)...” (27, p. 8). This issue of timing is important. Among a cohort of hypertensive Chinese participants, large differences in the amount of bias produced by the Kawasaki formula were observed when the second morning urine was used as compared with a late afternoon or early evening sample (32). Additionally, Mente et al. included participants with a wide range of 24-hour sodium excretion (mean = 4,116 mg/day; range, 849–13,248 mg/day). It is possible that the INTERSALT and Tanaka equations produce less group-level bias within a less extreme range of sodium excretion, such as in our study (mean was 3,300 mg/day) and prior studies (2–6). In Mente's analysis, it appears that the Kawasaki equation produces somewhat less underestimation (fewer individuals outside the 95% limits of agreement) at high levels of sodium excretion, compared with the INTERSALT and Tanaka equations (4). When extremely high values of sodium excretion are included, as in Mente's study, these outliers could pull down the average estimated bias for all prediction equations, resulting in an underestimation in the INTERSALT and Tanaka equations and little to no average bias in the Kawasaki equation. It is possible that in Mente's study, excluding outlying sodium values might produce results more consistent with our findings; at present, the reasons for these differences remain unclear.

Our study has several strengths, including a large and diverse population of older men and women with 24-hour fractional urine collection and detailed phenotyping, including physical examination, medication use, and dietary consumption. Further, 24-hour urine specimen collection started and stopped at the study center to ensure accurate timing of collection. While this population comprises older adults in a single US city, the proportion with hypertension and antihypertensive medication use is similar to that of the US population as a whole. By design, we overenrolled African Americans in order to assess racial/ethnic differences as well as differences in the validity of the predictive equations.

There are however, several limitations to consider. There is the potential for selection bias; however, our study participants had similar demographic characteristics, although a slightly higher proportion of African Americans, compared with all eligible individuals from MESA and CARDIA. These findings among MESA and CARDIA participants may not be generalizable to the entire US population. The completeness of the 24-hour urine collection was assessed by questionnaire. Among our participants, only 2% reported missing a void, and none missed more than one, possibly because we started and stopped urine collection at the study center. We did several sensitivity analyses to examine the potential impact of completeness on our findings—including omitting individuals with low volume, those who reported missing any voids or urine during collection, and those who had low observed-to-expected creatinine ratio—and our findings remained consistent.

This study extends our understanding of the ability of timed spot urine specimens to predict 24-hour sodium excretion among a population of older adults, over half with hypertension. A single spot urine is not a valid estimate of individual intake. The use of a timed spot urine specimen could potentially represent a feasible and cost-effective approach for monitoring national sodium intake; however, the use of previously published equations to estimate the mean 24-hour sodium excretion from a single timed spot specimen may be problematic, given that all 4 published equations tested in this study resulted in significant over- or underestimation of 24-hour sodium excretion in older, hypertensive, African-American and white men and women. The Tanaka equation using the evening sample produced the least bias overall, but the amount of bias varied significantly and dramatically by sex-specific and racial/ethnic subgroups. Further analyses, including evaluating the use of more than one spot urine specimen to capture a greater amount of total sodium excretion, sex- and race/ethnicity-specific equations, and the adjustment of within-person day-to-day variability, are needed in the utilization of timed spot samples to monitor population sodium intake among older adults and link individual levels with outcomes.

## Supplementary Material

Web Material
