# Supplementary material for: The Validity of Predictive Equations to Estimate 24-Hour Sodium Excretion: The MESA and CARDIA Urinary Sodium Study
Source: Am J Epidemiol. 2017 Jun 9;186(2):149–59. doi: 10.1093/aje/kwx056 (PMC5860382; doi:10.1093/aje/kwx056)
Supplement: Web Material [file kwx056allenwebmaterialfinal.pdf]

WEB MATERIAL

Web Table 1. Demographics for participants in the study compared with all eligible participants

	USS study participants	Eligible non-participants	p-value[1]
Sample size, n[2]	554	568	
Female, n (%)	310 (56.0)	317 (55.8)	0.9605
Age, y	60.3 ± 9.1	57.2 ± 9.8	0.3075
Black, n (%)	319 (57.6)	261 (46.0)	<.0001
Hypertension, n (%)	300 (54.2)	230 (40.8)	0.5485
BMI, kg/m^2	30.0 ± 6.5	29.4 ± 6.7	0.4375

[1]Chi square or t-test p-values, as appropriate, comparing characteristics of USS study participants with eligible non-participants

[2] 47.7% CARDIA, 41.7% MESA, 10.7% MESA Family for participants;
48.9% CARDIA, 37.3% MESA, 13.7% MESA Family for non-participants

Web Table 2. Predictive equations for 24-h sodium excretion based on single spot urinary sodium concentrations[1]

Equation/ Reference	Spot Urine Collection Timing	Age range (y)	Low to high group mean observed 24-h sodium excretion[1]	Predictive Equation for 24-Hour Na Excretion (mg)
INTERSALT[2] Male (n=241) Female (n=2,852)	Random before 24-hour urine collection	20-59	3386–5520	Men: = 23 x {25.46 + [0.46 x spt NA (mmol/L)]- [2.75 x spot Cr (mmol/L)]-[0.13 x spot K (mmol/L)]+[4.10 x BMI (kg/m^2)] + [0.26 x age (yr)]}<br><br>Women: = 23 x {5.07 + [0.34 x spt NA (mmol/L)]- [2.16 x spot Cr (mmol/L)]-[0.09 x spot K (mmol/L)]+[2.39 x BMI (kg/m^2)] + [2.35 x age (yr)]-[0.03 x age^2 (yr)]}
Tanaka[3] (n=591)	Random before 24-hour urine collection	20-59	2709–3852	=23 x (21.98 x $XNa^{0.392}$), where XNA = [spot Na (mmol/l) / spot Creatinine (mg/dL) x 10] x Pr24hCr (mg/day)]<br><br>Pr24hrCr (mg/day) = [-2.04 x age (y)]+ [14.89 x WT (kg)] + [16.14 x HT (cm)]-2244.45
Kawasaki[4] (n=159)	2nd morning urine collection after 24-hr collection	20-59	3337–5150	=23 x (16.3 x $XNa^{0.5}$), where XNA = [spot Na (mmol/l) / spot Creatinine (mg/dL) x 10] x Pr24hCr (mg/day)]<br><br>M: Pr24hrCr (mg/day) = [-12.63 x age (y)] + [15.12 x WT (kg)] + [7.39 x HT (cm)]-79.9<br><br>F: Pr24hrCr (mg/day) = [-4.72 x age (y)] + [8.58 x WT (kg)] + [5.09 x HT (cm)]-74.5
Mage[5,6]	Random Urine Specimens	20-79	4287–5350	={[23 x spot NA (mmol/L)]/[spot Cr (mg/dL) x 10]} x [Pr24HrCr (mg/day)]<br><br>M: Pr24HrCr (mg/day) = 0.00179 x [140 – age (yr)] x [WT $(kg)^{1.5}$ x HT $(cm)^{0.5}$] x [1 + 0.18 x B x [1.366 – 0.0159 x BMI (kg/m^2)]<br><br>F: Pr24HrCr (mg/day) = 0.00163 x [140 – age (yr)] x [WT $(kg)^{1.5}$ x HT $(cm)^{0.5}$] x [1 + 0.18 x B x [1.429 – 0.0198 x BMI (kg/m^2)]

[1]Cogswell et al 2013 (Am J Clin Nutr)
[2] Brown et al 2013 (Am J Epi). INTERSALT, International Cooperative Study on Salt and Blood Pressure.
[3]Tanaka et al 2002 (J Human Htn)
[4]Kawasaki et al 1993 (Clin Exp Pharm & Phys)
[5]Mage et al 2008 (J Exp Sci & Env Epi)
[6]Huber et al 2007 (J Exp Sci & Env Epi)

Web Table 3. Characteristics of Day 1 urine collections[1]

	Total (n=554)
Voids collected per person	8.9 (3-23)
Total urine volume, mL	1838.3 ± 810.0 (512-5601)
Total collection time, h	23.9 ± 1.1
Missed one void during collection	12 (2.2)
Total timed-spot specimens collected, n	
Morning	540 (97.5)
Afternoon	528 (95.3)
Evening	545 (98.3)
Overnight	553 (99.8)
Timed-spot specimens provided by participants, n	
2	3 (0.5)
3	449 (7.9)
4	507 (91.5)

[1]Values are means (range), means ± SDs (range), or n (%).

Web Table 4. Distributions of total urine volume and 24-h excretion of sodium and creatinine in the day 1 collection by race and gender.[1]

			Percentile		
	N	Means ± SDs	25th	50th	75th
Total volume, ml					
Black male	134	1691 ± 731	1089	1566	2132
Black female	185	1727 ± 780	1143	1573	2245
White male	110	2053 ± 832*	1424	1907	2519
White female	125	1965 ± 865*	1393	1897	2313
Sodium, mg					
Black male	134	3454 ± 1651	2350	3237	4388
Black female	185	3397 ± 1641	2334	3168	4203
White male	109	3926 ± 1623*	2928	3795	4651
White female	124	2480 ± 1079*	1814	2245	3260
Creatinine, mg					
Black male	134	1961 ± 645	1526	1947	2278
Black female	185	1404 ± 456	1106	1383	1546
White male	109	1915 ± 526	1575	1877	2182
White female	124	1101 ± 263*	921	1068	1249

[1]Values are means ± SDs or percentiles.
*Within an analyte, different from blacks within the same gender, $p < 0.05$

Web Table 5. Distributions of mean urine volume and concentration of sodium, and creatinine in timed-spot urine specimens in the day 1 collection by race and gender.[1,2,3]

	Timing of spot urine collection			
	Overnight	Morning	Afternoon	Evening
Total volume, ml				
Black male	290 ± 125	190 ± 114	196 ± 99	192 ± 100
Black female	325 ± 182	160 ± 115	161 ± 104	183 ± 109
White male	353 ± 184*	219 ± 147	226 ± 112*	226 ± 110*
White female	371 ± 165*	193 ± 156*	193 ± 128*	194 ± 120
Sodium, mmol/L				
Black male	96 ± 55	106 ± 54	109 ± 53	110 ± 56
Black female	89 ± 55	107 ± 60	111 ± 58	110 ± 59
White male	97 ± 46	92 ± 49*	96 ± 52	101 ± 51
White female	67 ± 42*	77 ± 48*	70 ± 49*	74 ± 47*
Creatinine, mmol/L				
Black male	13 ± 8	13 ± 8	13 ± 8	16 ± 9
Black female	9 ± 6	10 ± 7	11 ± 8	13 ± 7
White male	11 ± 6*	10 ± 6*	10 ± 5*	12 ± 6*
White female	6 ± 4*	7 ± 5*	8 ± 6*	9 ± 6*

[1]Values are means ± SDs or percentiles.
[2]Timing of specimens: overnight (the first void after the longest period of sleep), 0400–1200 h; morning (the second void upon rising in the morning), 0830 to 1230 h; afternoon, 1231 to 1730 h; and evening, 1731 to 2359 h.
[3]Includes 134 Black men, 185 Black women, 110 White men and 125 White women

Web Table 6. Within-person, day-to-day CVs and the ratio of within- to between-person variance of sodium, potassium, chloride, creatinine, and iodine excretions in 24-h urine samples and timed-spot urine specimens[1,2,3]

	Within-person CV, %					Ratio of within- to between-person variance				
	24-h	Overnight	Morning	Afternoon	Evening	24-h	Overnight	Morning	Afternoon	Evening
Sodium, mg										
Black	37.9	56.3	70.3	64.3	55.3	1.5	2.4	4.0	4.5	1.8
White	31.3	41.4	52.2	52.3	53.0	1.1	0.7	1.1	0.9	1.8
Creatinine, mg										
Black	24.4	41.0	51.0	48.7	46.5	0.8	1.1	2.1	1.8	1.8
White	51.2	33.6	40.8	42.3	47.1	2.9	0.5	1.2	0.8	1.0

[1]Within-person CVs were calculated as the square root of the within-person variance divided by the mean of each analyte.
[2]Only included individuals with two day urine collections.
[3]Includes 48 Black men, 63 Black women, 35 White men and 40 White women

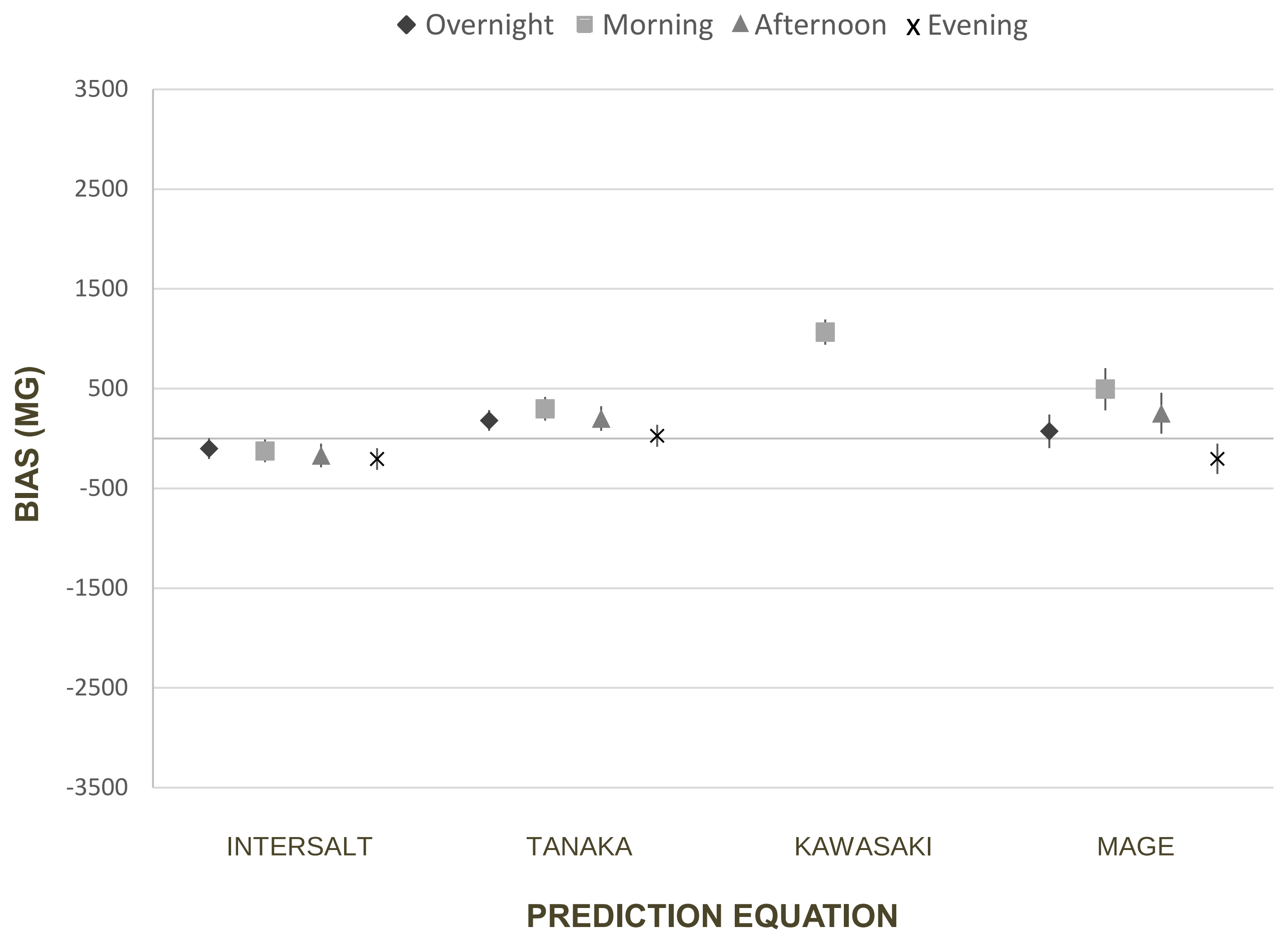

Web Figure 1. Mean bias in predicted minus measured 24-h sodium excretion based on the same day by prediction equation and by timing of the spot urine collection in adults. The mean bias in predicted 24-h sodium excretion by using each equation is shown for a single urine specimen collected at various time-spots. The 95% CIs for the mean bias are shown by the vertical lines surrounding each mean. INTERSALT, International Cooperative Study on Salt and Blood Pressure. n=554

Web Table 7. Predicted 24-h Sodium Excretion and Mean Bias Produced by using the Equation with the Least Bias for Each Gender-Race Group[1]

		Measured 24-h sodium excretion		Predicted 24-h urinary sodium excretion (mg/d)		Bias (Predicted – Measured) in 24-h urinary sodium excretion		
	n	Mean	SD	Mean	SD	Bias	Lower CI	Upper CI
Overnight	553	3289.1	1552.2	3329.0	1322.6	41.5	-71.9	154.8
Morning	540	3320.3	1611.8	3479.0	1386.2	160.2	29.6	290.8
Afternoon	528	3335.9	1610.8	3366.2	1351.3	34.2	-97.4	165.8
Evening	545	3319.1	1604.3	3206.4	1308.7	-111.2	-229.1	6.7

[1]Across all times, the Tanaka equation was used for black males and black females, INTERSALT equation for white males, Mage equation for white females.

Web Table 8. Intraclass Correlation Coefficients With Measured 24-hour Sodium Excretion, by Population Subgroup and the Timing of Spot Urine Specimen Collections in Adults Aged 45–79 Years, Chicago, Illinois, 2013[a]

	n	Predicted 24-h Sodium Excretion			
		INTERSALT	Tanaka	Kawasaki	Mage
All					
Overnight	540	0.54	0.51		0.56
Morning	529	0.50	0.44	0.36	0.41
Afternoon	520	0.48	0.42		0.43
Evening	537	0.54	0.51		0.58
Black men					
Overnight	131	0.51	0.55		0.59
Morning	124	0.43	0.48	0.4	0.5
Afternoon	119	0.36	0.33		0.41
Evening	128	0.52	0.49		0.54
Black women					
Overnight	179	0.4	0.57		0.51
Morning	180	0.32	0.33	0.24	0.22
Afternoon	170	0.31	0.38		0.30
Evening	181	0.41	0.55		0.60
White men					
Overnight	108	0.36	0.39		0.53
Morning	105	0.42	0.52	0.51	0.62
Afternoon	108	0.33	0.44		0.57
Evening	109	0.38	0.41		0.51
White women					
Overnight	122	0.53	0.35		0.54
Morning	120	0.52	0.32	0.18	0.49
Afternoon	123	0.51	0.37		0.51
Evening	119	0.54	0.43		0.55

[a]All values are intraclass correlation coefficients between measured 24-hour sodium excretion and predicted 24-hour sodium excretion based on sodium concentration from a spot urine collection used with 1 of the 4 estimation equations: INTERSALT, Tanaka, Kawasaki, and Mage. The timing of urine specimens was as follows: overnight (the first void after the longest period of sleep), 0400–1200 the next morning; morning (the second void on rising in the morning), 0830–1230; afternoon, 1231–1730; and evening, 1731–2359. All intraclass correlation coefficients were statistically significant at P<0.05. INTERSALT, International Cooperative Study on Salt and Blood Pressure.

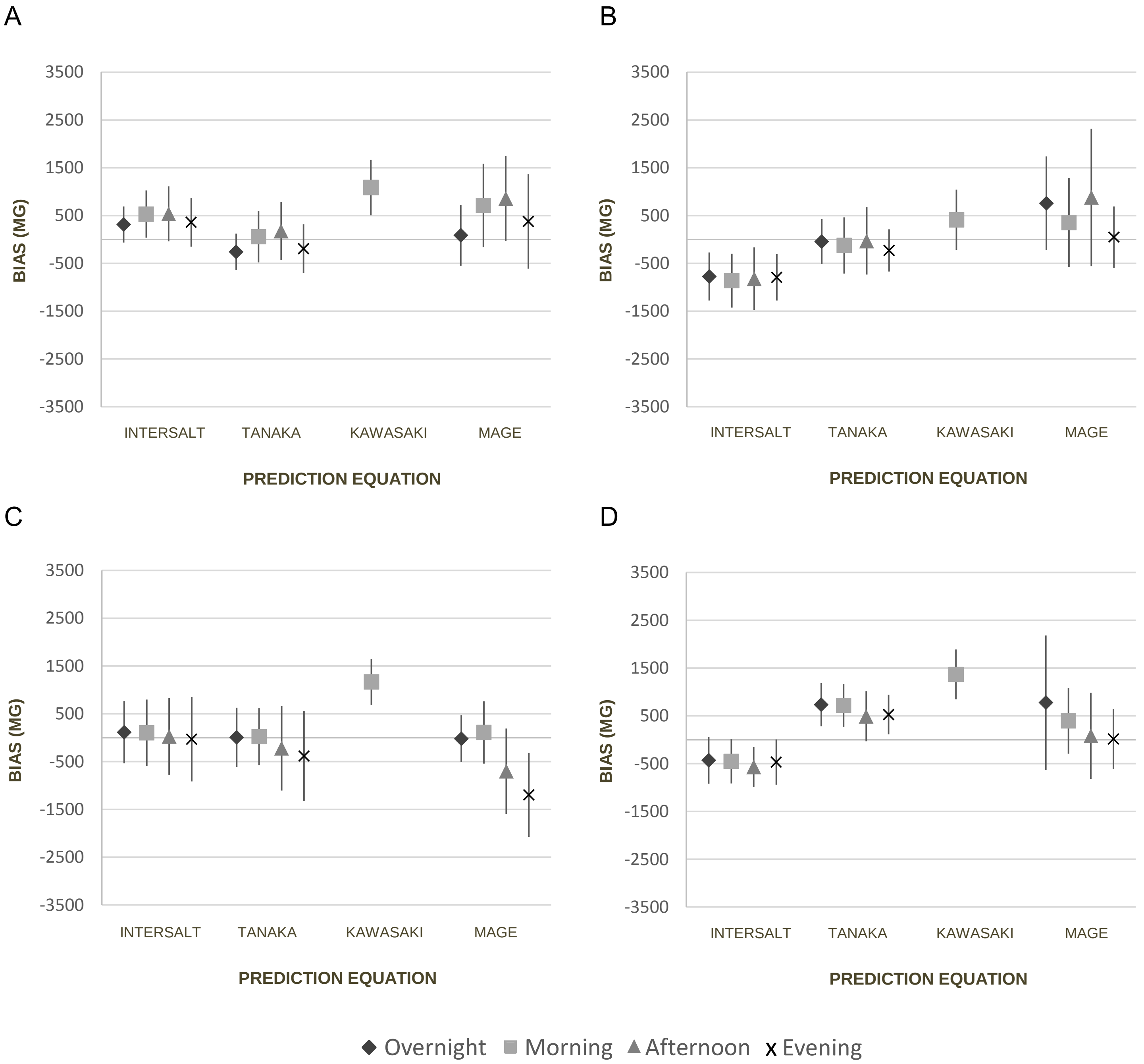

Web Figure 2. Mean bias in predicted minus measured 24-h sodium excretion based on the same day by prediction equation and by timing of the spot urine collection in adults within the following race-sex subgroups: black men (A) (n=32), black women (B) (n=42), white men (C) (n=14), and white women (D) (n=17) for participants with **uncontrolled hypertension**. The mean bias in predicted 24-h sodium excretion by using each equation is shown for a single urine specimen collected overnight, in the morning, in the afternoon, and in the evening. The 95% CIs of the mean bias are shown by the vertical lines surrounding each mean. Total N=105.

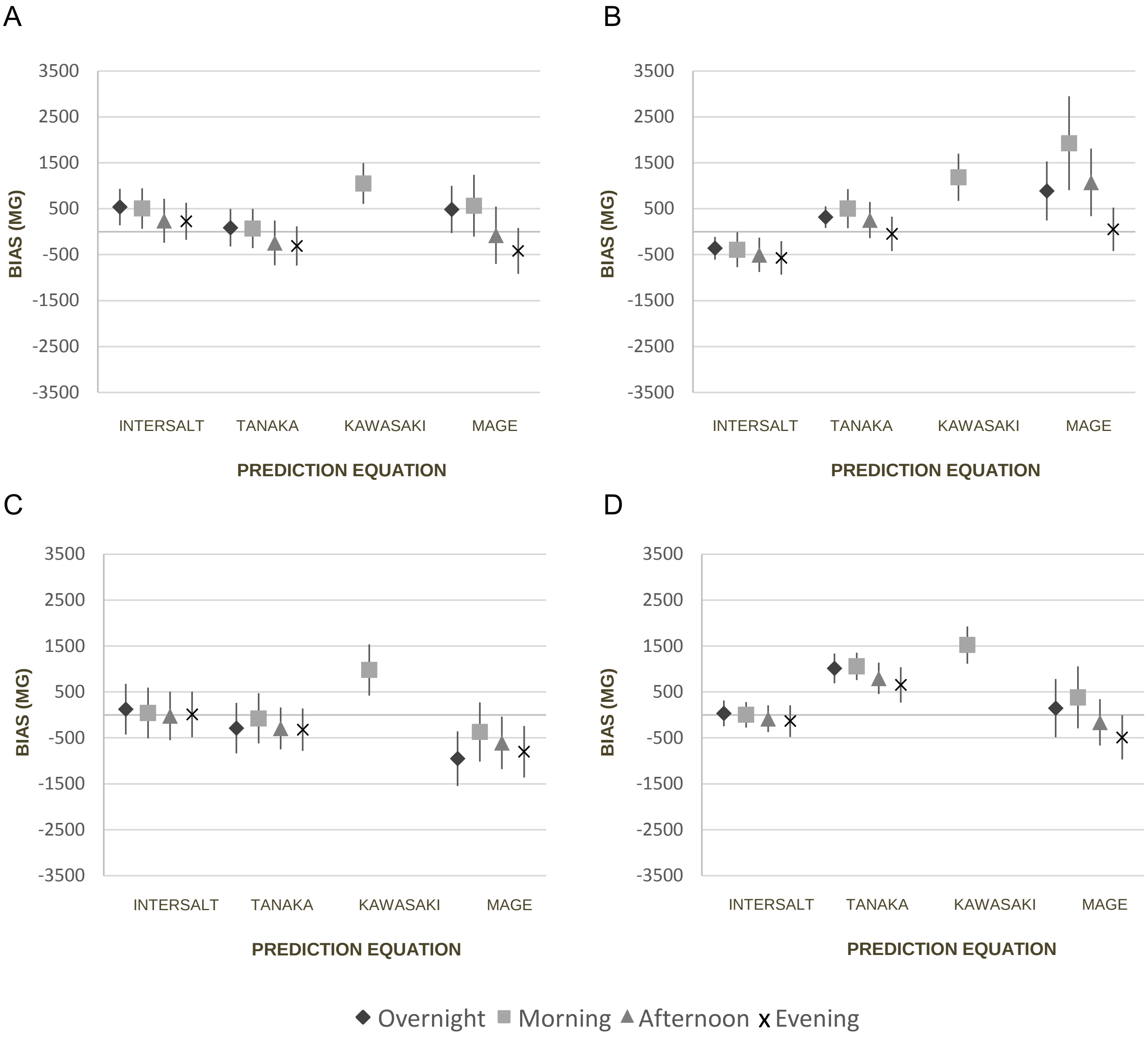

Web Figure 3. Mean bias in predicted minus measured 24-h sodium excretion based on the same day by prediction equation and by timing of the spot urine collection in adults within the following race-sex subgroups: black men (A) (n=44), black women (B) (n=64), white men (C) (n=38), and white women (D) (n=32) for participants with **controlled hypertension**. The mean bias in predicted 24-h sodium excretion by using each equation is shown for a single urine specimen collected overnight, in the morning, in the afternoon, and in the evening. The 95% CIs of the mean bias are shown by the vertical lines surrounding each mean. Total N = 178.

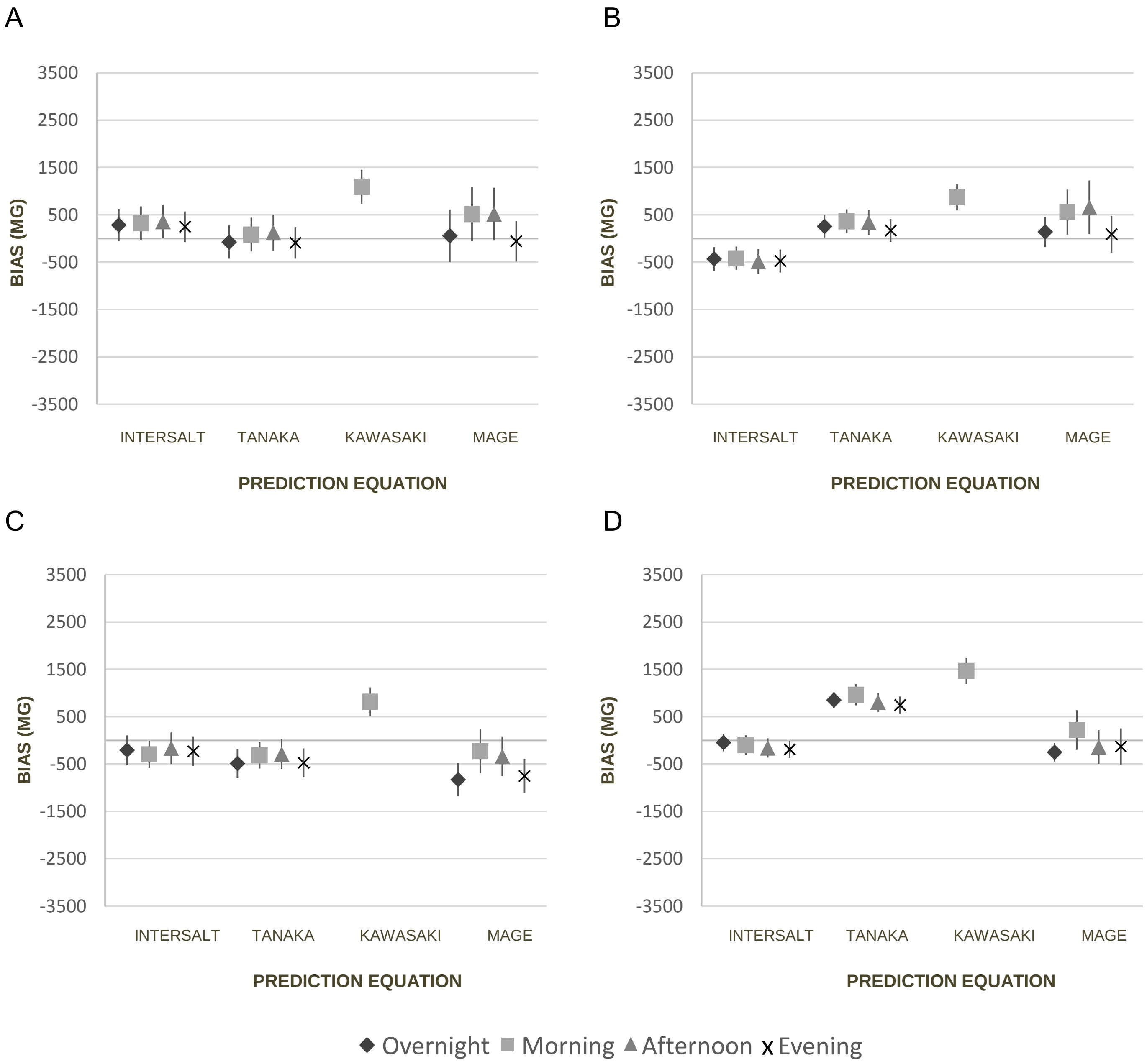

Web Figure 4. Mean bias in predicted minus measured 24-h sodium excretion based on the same day by prediction equation and by timing of the spot urine collection in adults within the following race-sex subgroups: black men (A) (n=58), black women (B) (n=79), white men (C) (n=58), and white women (D) (n=76) for participants with **no hypertension**. The mean bias in predicted 24-h sodium excretion by using each equation is shown for a single urine specimen collected overnight, in the morning, in the afternoon, and in the evening. The 95% CIs of the mean bias are shown by the vertical lines surrounding each mean. Total N = 271.

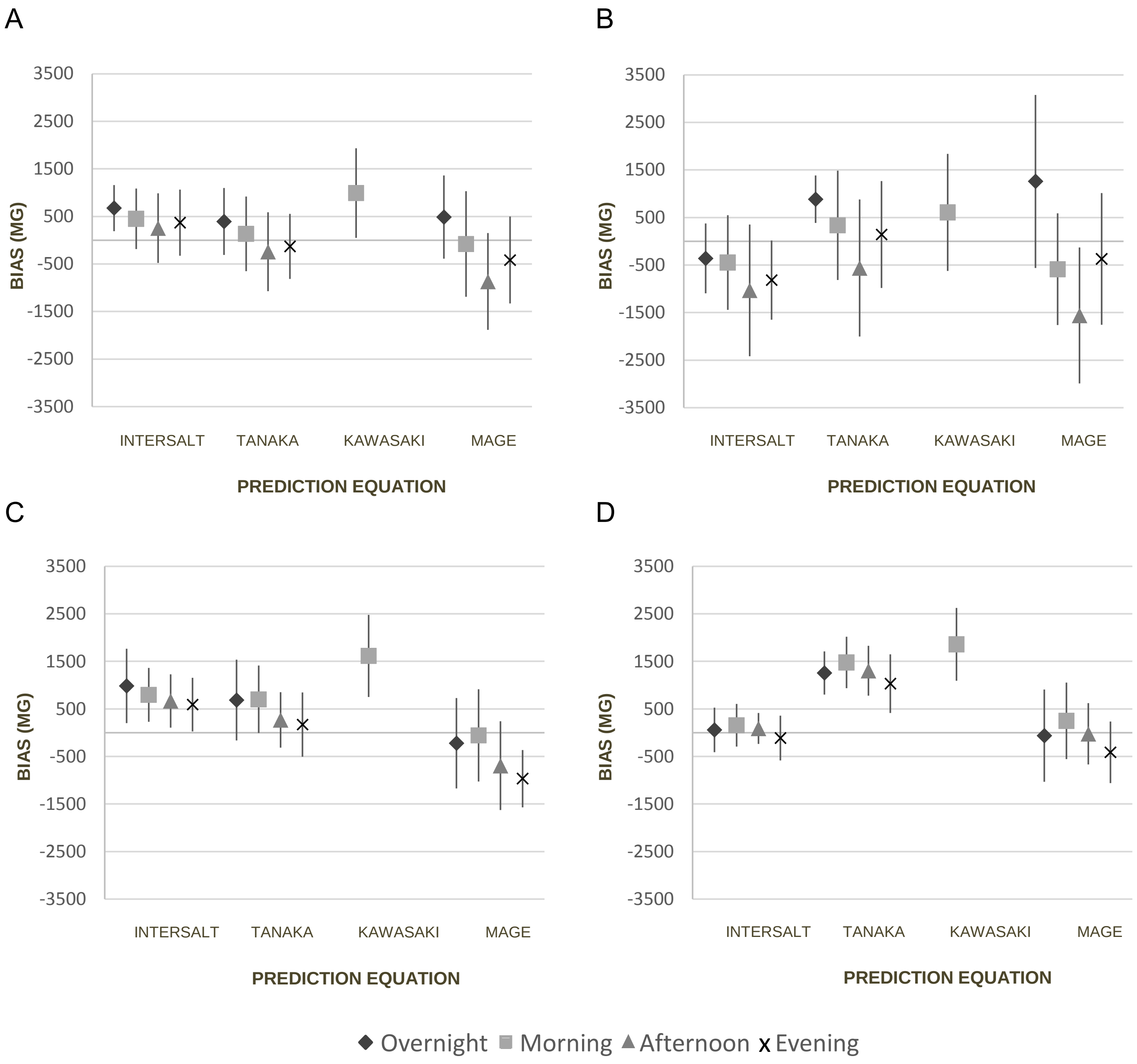

Web Figure 5. Mean bias in predicted minus measured 24-h sodium excretion based on the same day by prediction equation and by timing of the spot urine collection in adults within the following race-sex subgroups: black men (A) (n=10), black women (B) (n=9), white men (C) (n=8), and white women (D) (n=9) for participants with **eGFR≤60**. The mean bias in predicted 24-h sodium excretion by using each equation is shown for a single urine specimen collected overnight, in the morning, in the afternoon, and in the evening. The 95% CIs of the mean bias are shown by the vertical lines surrounding each mean. Total N = 36.

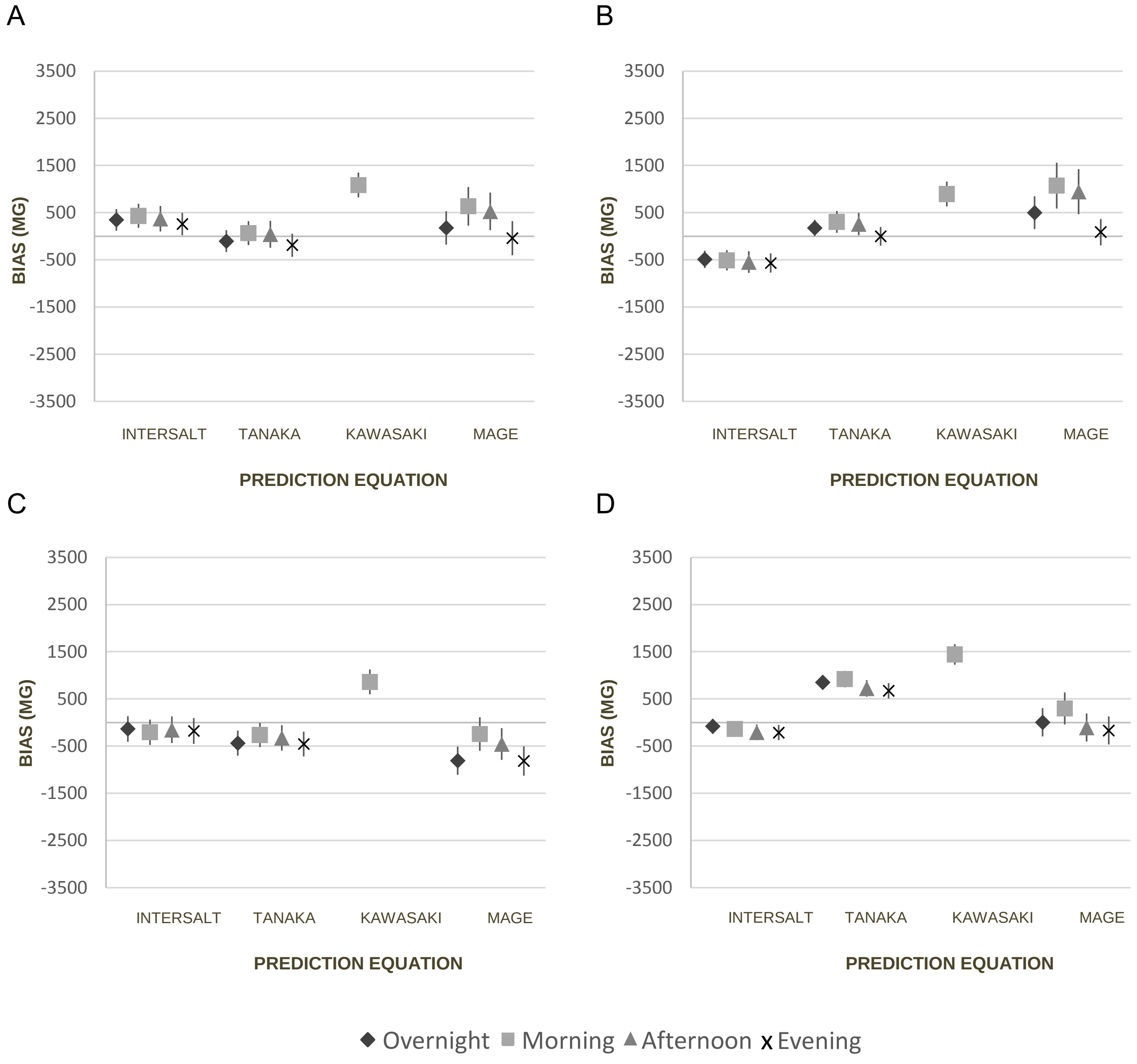

Web Figure 6. Mean bias in predicted minus measured 24-h sodium excretion based on the same day by prediction equation and by timing of the spot urine collection in adults within the following race-sex subgroups: black men (A) (n=124), black women (B) (n=176), white men (C) (n=102), and white women (D) (n=116) for participants with **eGFR>60**. The mean bias in predicted 24-h sodium excretion by using each equation is shown for a single urine specimen collected overnight, in the morning, in the afternoon, and in the evening. The 95% CIs of the mean bias are shown by the vertical lines surrounding each mean. Total N = 518.
